# Opinions of Gynecologists About Indication and Technique of Perineoplasty

**DOI:** 10.3390/jcm13247536

**Published:** 2024-12-11

**Authors:** Esther C. A. M. van Swieten, Karlijn J. van Stralen, Astrid Vollebregt, Jan-Paul W. R. Roovers

**Affiliations:** 1 Department of Obstetrics and Gynaecology, Spaarne Gasthuis, 2035 RC Haarlem, The Netherlands; kvanstralen@spaarnegasthuis.nl (K.J.v.S.); avollebregt@spaarnegasthuis.nl (A.V.); 2 Department of Obstetrics and Gynaecology, Amsterdam UCM, 1105 AZ Amsterdam, The Netherlands

**Keywords:** perineoplasty, perineorrhaphy, indications, surgical technique, survey, prolapse, genital hiatus

## Abstract

**Background.** Perineoplasty is a frequently performed procedure as part of prolapse surgery. Despite its frequent use, there is a lack of evidence on the optimal indication, surgical technique and adverse outcomes. We intended to gain insight into the current opinions on indications and techniques of perineoplasty among (uro)gynecologists worldwide. **Methods.** We conducted a survey among members of the International UroGynecological Association (IUGA) to objectify indications for perineoplasty and aspects of surgical technique. **Results.** A total of 114 urogynecologists responded, with 98% performing perineoplasty. A total of 85% of respondents aimed to approximate the bulbocavernosus muscle, whereas 27% aimed to include the puborectal muscle as well. A total of 86% of respondents used 1–4 resorbable sutures, especially vicryl 2/0 (39%) or vicryl 0 (52%). According to the respondents, a “wide genital hiatus at physical examination” (87%) and “subjective complaints of a wide genital hiatus” (84%) were considered good/excellent indications for perineoplasty, whereas “fecal incontinence”, “apical prolapse” and “perineal pain” were absolutely/mostly not a good indication. Reasons to not perform perineoplasty were pelvic pain (59%) and dyspareunia (64%). Most responders underlined the need for more research on this topic (8.5 out of 10). **Conclusions.** Perineoplasty is a frequently performed procedure. There is a wide variation in the indications for and surgical techniques of perineoplasty. Therefore, research is needed to identify which patients will benefit from perineoplasty and how to optimally perform this surgery.

## 1. Introduction

Perineoplasty, also referred to as perineorrhaphy, is a commonly performed procedure during pelvic organ prolapse (POP) surgery [1]. It is often combined with other vaginal or abdominal prolapse operations such as anterior and/or posterior colporrhaphy, sacrospinous fixation or sacrcocolpopexy but can also be performed as a stand-alone procedure.

Perineoplasty implies surgical repair of the perineum or perineal body [2]. It aims to restore level III support according to deLancey [3] and to reduce the size of the genital hiatus (GH). Nevertheless, the ideal indication criteria, when to withdrawal from the procedure, what anatomical structures should be involved, what suture materials should be used, the optimal number of sutures and other surgical aspects are not clearly described.

In recent years, there has been evidence that an enlarged GH (which results in a lack of adequate support for the pelvic organs) is associated with the development of pelvic organ prolapse [4]. In addition, a wider GH after prolapse surgery was found to be an independent risk factor for recurrent POP or anatomic failure at follow-up [5,6,7,8,9]. Incorporating perineoplasty into prolapse surgery might provide additional support to the pelvic structures and improve the overall outcome. This extended support could potentially reduce the likelihood of recurrent prolapse and the need for re-intervention.

Furthermore, perineoplasty is sometimes performed with the aim of reducing urinary and fecal incontinence. Since the perineum plays a role in controlling the opening and closing of the urethra and anus, reinforcement in the case of perineal descent or weakness might contribute to voluntary control over urination and defecation [10,11,12]. In the case of a grade four obstetric perineal laceration, the functional outcome seems better when repairing the obstetric laceration is combined with performing a perineoplasty [13].

Moreover, the perineum is involved in sexual function, with its muscles and connective tissue contributing to vaginal tone and sensation. The perineal muscles play a role in sexual arousal and orgasm, highlighting the intricate relationship between the perineum and sexual health. A study by Dogan [14] described that surgical treatment of stress urinary incontinence (SUI) improves sexual function and that this effect might be stronger when adding a perineoplasty in patients with perineal defect and vaginal enlargement. The procedure can be performed as a stand-alone surgery and to improve sexual function [15,16,17,18,19].

Finally, some small studies suggest that perineoplasty is indicated for subjective symptoms related to a wide GH, like the sensation of a wide or open vagina, decreased friction during coitus or water or air going in and out of the vagina during bathing or intercourse [15,17,20].

As shown above, the indications for performing a perineoplasty are not agreed upon and are widely debated and heterogeneous. As there is clear evidence that addressing level III support results in better anatomical and functional outcomes, we intend to further knowledge on perineoplasty. We explored the opinions of urogynecologists regarding the indications to perform a perineoplasty and asked them to rank the importance of these indications. Secondly, we described the variations in surgical technique among urogynecologists worldwide.

## 2. Materials and Methods

We conducted a web-based anonymous survey amongst all members of the International Urogynecological Association (IUGA). The survey was sent by email to IUGA members and was open to be completed from June to December 2023. During this period, a total of three dedicated mailings about the survey were sent, there were two social media posts about it, the survey was visible on the IUGA website and it was mentioned in two general mailings of the IUGA.

The survey was tested before release on a panel of four (uro)gynecologists working in different hospitals and settings.

The survey included a total of 23 questions. Surgeons’ work experience and training were queried as were details about their surgical practice (size and characteristics). We asked in detail about the surgical technique that was used when performing a perineoplasty. This included questions about the excision of tissue, which muscles and structures are involved and the amount and type of sutures that are used. We identified nine indications to perform a perineoplasty and asked the respondents to rate these indications on a scale ranging from “absolute not a good indication” to “excellent indication” (converted to a 0–5 scale). The same approach was applied to five reasons for withdrawing from performing a perineoplasty, ranging from “absolutely not a reason to withdrawal” to “excellent reason to withdrawal”. Details about the GH size at which a perineoplasty should be performed were queried. Furthermore, we questioned them regarding their opinion of the scientific or clinical evidence for performing a perineoplasty and about how relevant is it to perform further research on the (possible) added value of perineoplasty on a scale of 1–10. At the end of the questionnaire, there was space for free text on the topic.

We presented the results as percentages per group and means with SD. Means were calculated using the lowest category as 0 and the highest as 4.

### Statistical Analysis

We performed ordinal generalized linear modeling to study factors associated with indications for performing perineoplasty and reasons not to perform this procedure. We included the years working as a specialist (reference 1–5 years), type of additional urogynecological training (reference formal training), number of new patients per year per surgeon at the outpatient clinic (reference < 400/year), number of POP surgeries per year (reference < 100/year) and region (reference = Europe) as categorical variables and did not adjust for confounding factors. A factor was considered to be associated with the outcome if the p-value was below 0.05.

## 3. Results

A total of 114 urogynecologists from 43 different countries completed the survey. Of them, 61% have been a specialist for more than 10 years, and 21% for less than 5 years. The majority underwent a formal subspecialty training (57%). A majority of specialists (59%) treat less than 400 new prolapse patients per year. Regarding the surgical volume, half of them (50%) perform more than 100 procedures for prolapses per year (procedures for stress urinary incontinence not included), with 4% performing more than 250 procedures per year. With respect to the characteristics of their surgical practice, 94% regularly perform vaginal surgery, with 76% performing mainly vaginal procedures and less laparoscopic or robotic ones (Table 1).

Virtually all respondents (98%) sometimes perform perineoplasty during prolapse surgery. When characterizing the surgical technique, 85% re-approximates the bulbocavernosus muscle. We observed a variety regarding the amount of sutures used; 53% indicate that they use mostly one or two sutures, while 33% use three to four sutures. With respect to the kind of sutures, slightly more than half (52%) use type 0 sutures and 39% usually use type 2/0. Performing a perineoplasty is often part of prolapse surgery and is seldom performed as a stand-alone procedure. Almost all surgeons (91%) usually excise tissue during the procedure, of which 74% individualizes the amount of excised tissue.

Regarding the question about at what hiatal size a perineoplasty could be considered, we observe that there is agreement that at a GH of less than 4 cm, there is no indication for perineoplasty while at a GH of greater than 8 cm, the majority of respondents would definitely perform one. On the other hand, there is no consensus on what would then be a good cut-off value; about 50% believe that an intervention should be performed at a GH from 4 cm onwards, while 42% believe this should only be an issue from 6 cm onwards (Table 2).

When looking at the indications to perform perineoplasty, overall, the two most important indications for performing a perineoplasty are “wide genital hiatus at physical examination by POP-Q” and “subjective complaints of the patient of a wide genital hiatus (feeling of being open)”. Responders from Africa consider “wide genital hiatus at physical examination” to be a significantly less good indication to perform a perineoplasty than surgeons from other continents. Moreover, for responders from North America “subjective complaints of vaginal flatus” is a significantly less common reason to perform perineoplasty than for surgeons elsewhere. “Subjective complaints of patients partner of insufficient friction during intercourse” is considered by the majority of the responders as not a good indication for the procedure, as is fecal incontinence, except by surgeons from Oceania and Africa, who assess this complaint in advance of performing a perineoplasty. Finally, surgeons who are more hesitant to perform a perineoplasty because of perineal skin problems were less likely to have formal training, and surgeons who perform more prolapse surgeries per year are more hesitant to perform perineoplasty because of perineal pain (Table 3).

The main reasons to refrain from perineoplasty during prolapse surgery are current pelvic pain and current dyspareunia. The fear of developing one of these two is also listed as a reason to not perform the procedure, but surgeons from South America and responders with longer working experience do not consider the “fear of developing dyspareunia” as a reason to refrain from perineoplasty. A prolonged time of surgery is not a reason to not perform perineoplasty when a surgeon thought it was indicated (Table 4).

There is a difference in views regarding the scientific and clinical evidence for performing a perineoplasty. Forty-six percent of the respondents believe there is scientific evidence, while eighty-nine percent of surgeons report clinical evidence for performing the procedure.

The relevance of further research on the (possible) added value of perineoplasty was rated with an 8.5 on a 1–10 scale by the respondents.

In reaction to the free text space, there were mainly remarks on either the need for further description of the technique like “we should describe much more accurately the technique” and “because perineorrhaphy is performed with such variability, research has to really qualify what is meant by the term to be helpful” or the endorsement of the clinical or scientific evidence, like “a wide hiatus is a reason for recurrence of POP as DeLancey showed in his studies”, “If properly performed it plays a tremendous role in psychological satisfaction of the patient along with relief of physical symptoms” and “very good topic that will generate very useful information”.

## 4. Discussion

In our survey, we found that a vast majority of urogynecologists (89%) consider clinical evidence available to justify the performance of a perineoplasty during prolapse surgery and that this procedure belongs within the pallet of procedures regarding the surgical treatment of prolapses. Nevertheless, there seems to be no consensus about when to perform it, what patients could benefit from the procedure and how a perineoplasty should exactly be performed. This all reflects the lack of appropriate guidelines regarding when and how to perform perineoplasty.

The most important reason to perform a perineoplasty was “a wide genital hiatus at physical examination”. This is consistent with the literature describing that there appears to be a protective effect of a smaller GH on the development of prolapse [4,21] and that a wider GH increases the risk of recurrent prolapse after surgical repair [1,5,6,7,8,9,22,23]. Bonglack et al. [24] describe that the reduction in the GH due to perineoplasty sustains after surgery, but their follow-up was limited to three months. This was also reported by Mothes [25], who describes this persisting effect on GH post-operatively. However, to our knowledge, there is no known prospective study yet examining whether this reduction in the GH has a protective effect on the risk of recurrent prolapse.

Furthermore, when describing the GH at a POP-Q examination, one reflects what surgeons see at their physical examination and what is a representation of the GH at the skin level. In our opinion, this is the reason why the correlation between the GH at POP-Q and the effect of this GH on prolapse is limited. The level of the puborectal muscle is most likely more related to pelvic floor function, but can only be assessed based on palpation or imaging techniques. Since the GH in the POP-Q determination only measures the skin and no assessment is made of the underlying muscle and connective tissue complex (which in fact determines the support and function of the pelvic floor), the GH might not be the most reliable factor related to the (re)occurrence of prolapses. Further research should preferably focus on the measurement of the underlying muscle using techniques like (3D) ultrasound or MRI.

Taking a closer look at which patients could benefit from perineoplasty, the consideration “subjective complaints of a wide genital hiatus” was indicated as “sometimes good/excellent” by a large proportion (84%) of respondents, suggesting that this might be the group of women who benefit from this procedure. Performing a perineoplasty with the aim of tightening the vagina (diminishing the sensation of a wide vagina) is described in different studies [15,17,18], and this aspect is often combined with the complaint of “insufficient friction during intercourse”. In our survey about half of the responders (53%) considered sexual complaints of the patient as a good/excellent reason to perform a perineoplasty, while different studies [16,18] describe the improvement of sexual functioning in both the patient and partner (without an increase in the risk of dyspareunia) as a result of perineoplasty in patients with a subjective feeling of a wide vagina. In our survey, it appeared that urogynecologists considered performing perineoplasty only when the patient suffered insufficient friction and not if the partner experienced insufficient friction.

Fecal incontinence was seen as an indication of perineoplasty by a minority of responders (20%), which is confirmed by the paucity of literature on this topic. We only found studies that describe the effects of perineoplasty when combined with sphincter surgery after OASI or in case of malformations [13,26]. Nevertheless, the clinical experience of the authors is that fecal complaints can improve after the correction of the anatomy of the perineal body by performing a perineoplasty, so it might be hypothesized that perineoplasty has a positive effect on defecation symptoms. although this is not confirmed by the current literature, so further research on this topic might be useful.

In relation to the surgical technique, a majority of the respondents (85%) indicate suturing the bulbocavernosus muscle while only 27% extend to the puborectal muscle. A study by Salehi [26] reports a de novo dyspareunia rate of 53% after perineoplasty when involving the puborectal muscle as well. This seems a high percentage and thus restraint extending to the puborectal muscle might be considered. It is notable that the individualization of the procedure is important to surgeons, which represents the delicate balance between the possible positive effects of perineoplasty and adverse outcomes such as pain and dyspareunia. In addition, we believe it is essential to investigate and record exactly which anatomical structures should be involved in perineoplasty. Only by a detailed description of the surgical technique is it possible to study the anatomical and functional outcomes and generalize the findings of the studies to the rest of the world.

In a recent article, deLancey [27] presented a detailed description of the functional anatomy of the perineal complex based on both MRI and cadaver studies. He showed that the connections between levator ani muscles, perineal membrane, perinal body and vaginal fascia all have a role in the closure mechanism of the genital hiatus. This article could help to precisely describe a standard surgical technique when performing a perineoplasty with the aim of restoring hiatal closure. The next step would be to perform a prospective study in which patients undergoing surgery for prolapse with and without perineoplasty are compared in terms of surgical success, adverse outcomes, etc.

One of the strengths of our study is that by including urogynecologists from more than 40 countries, our findings are generalizable worldwide. Of course, there are some limitations: urogynecologists with an interest in perineoplasty are more motivated to participate in our survey. Furthermore, it is known that such surveys always have relatively low responder rates, but with the final number of responses, we feel that all relevant topics related to perineoplasty have adequately been addressed.

The free space that we included showed that we did not fail to include relevant topics and that colleagues share our opinion that level III support is relatively under-addressed in the existing scientific literature.

Given the variations in practice and the current lack of existing scientific evidence, there is a strong need for more information. There is a need for an (inter)national guideline on the scope of perineoplasty, which defines the role of this procedure Prospective research will reduce the uncertainty and heterogeneity between physicians and is needed to identify which patients will benefit from perineoplasty and how this surgery can be performed optimally.

## 5. Conclusions

This survey shows that perineoplasty is a procedure that pelvic floor surgeons frequently perform. There is a wide variation in the indications for and surgical techniques of perineoplasty. As a consequence, there is a need to research which patients will benefit from perineoplasty and how this surgical procedure can optimally be performed.

## Figures and Tables

**Table 1:** Baseline.

		N	%
specialty	(Uro)gynecologist	113	99
	Urologist	1	1
how many years working as a specialist	1–5 years	24	21
	5–10 years	21	18
	>10 years	69	61
additional urogynecological training	Formal training	65	47
	Non-formal training	35	31
	No additional training	14	12
how many new patients per year	<400	67	59
	400–800	43	38
	>800	4	4
how many pop surgeries per year	<100	57	50
	100–250	52	46
	>250	5	4
surgical practice	0–25 vaginal/75–100 laparoscopic or robotic	7	6
	Around 50% vaginal/around 50% lap/rob	21	18
	75–100 vaginal/0–25% lap/rob	68	75
region	Europe	39	34
	USA/Canada	24	21
	Latin America	18	16
	Middle East	8	7
	Africa	13	11
	Asia (except Middle East)	5	4
	Oceania	7	6

**Table 2:** Surgical technique.

		N
usually excises tissue at perineoplasty	Yes	103 (91.2)
	No	10 (8.8)
amount of tissue excise	As restrictive as possible	23 (20.2)
	Individualize	81 (71.1)
	As much as possible	0
	Do not excise tissue	6 (5.3)
intentionally narrowing the vagina	Always	6 (5.3)
	Often	20 (17.5)
	Depends on the case	77 (67.5)
	Seldom	8 (7.0)
	Never	2 (1.8)
intending to prevent narrowing the vagina	Always	31 (27.2)
	Often	22 (19.3)
	Depends on the case	52 (45.6)
	Seldom	6 (5.3)
	Never	2 (1.8)
approximate bulbocavernosus muscle	Yes	96 (85.0)
	No	17 (15.0)
how many sutures	1–2 sutures	52 (45.6)
	2–4 sutures	33 (28.9)
	>4 sutures	4 (3.5)
	Depends on the case	10 (8.8)
extend suturing to puborectal muscle	Yes	30 (26.5)
	No	83 (73.5)
kind of sutures	Usually 2/0	40 (39.2)
	Usually 0	53 (51.9)
	Other	9 (8.8)
combined with prolapse surgery	Always as a combination	37 (32.7)
	Often as a combination	51 (45.1)
	Depends on the case	24 (21.2)
	Seldom as a combination	1 (8.8)
	Never, always separate	0
at what size would you	≥2–4	4 (3.5)
	≥4	55 (48.2)
	≥6	47 (41.2
	≥8	5 (4.4)
scientific evidence	I do not know if there is any scientific evidence	30 (26.3)
	There is no scientific evidence to decide when it is indicated	25 (21.9)
	There is scientific evidence to be liberal	4 (3.5)
	There is scientific evidence to indicate some patients	49 (43.0)
	There is scientific evidence not to indicate	6 (5.3)
clinical evidence	I do not know if there is clinical evidence	9 (7.9)
	There is clinical evidence to be liberal	22 (19.3)
	There is clinical evidence to indicate some patients	79 (69.3)
	There is clinical evidence not to indicate	3 (2.6)
how relevant (score 0–100)	Median (SD)	85 (25)

**Table 3:** Reasons in favor.

	Absolutely Not N(%)	Mostly Not N(%)	Neutral N(%)	Sometimes Good N(%)	Excellent N(%)	Mean (SD)	Factors Affecting Risk
wide genital hiatus at physical	1 (0.9)	7 (6.1)	7 (6.1)	35 (30.7)	64 (56.1)	3.4 (0.9)	Africa ↓
subjective complaints wide genital hiatus	2 (1.8)	7 (6.1)	9 (7.9)	61 (53.5)	35 (30.7)	3.1 (0.9)	-
subjective complaints vaginal flatus	6 (5.3)	13 (11.4)	36 (31.6)	47 (41.2)	12 (10.5)	2.4 (1.0)	North America ↓
subjective complaints insufficient friction (patient)	7 (6.1)	26 (22.8)	21 (18.4)	49 (43.0)	11 (9.6)	2.3 (1.1)	-
subjective complaints insufficient friction (partner)	29 (25.4)	28 (24.6)	27 (23.7)	24 (21.1)	6 (5.3)	1.6 (1.2)	-
fecal incontinence	39 (34.2)	34 (29.8)	17 (14.9)	13 (11.4)	10 (8.8)	1.3 (1.3)	Oceania ↑ Africa ↑
mild apical prolapse	52 (45.6)	35 (30.7)	16 (14.0)	10 (8.8)	1 (0.9)	0.9 (1.0)	-
perineal pain	55 (48.2)	30 (26.3)	16 (14.0)	12 (10.5)	1 (0.9)	0.9 (1.1)	Number of surgeries ↓
perineal skin problems	36 (31.6)	28 (24.6)	26 (22.8)	20 (17.5)	4 (3.5)	1.4 (1.2)	No formal additional training ↓

↓ indicates that a factor is associated with negative outcomes such as “absolutely not a reason” or “mostly not a reason”.

**Table 4:** Reasons to refrain.

	Absolutely Not a Reason N(%)	Mostly Not N(%)	Neutral N(%)	Sometimes Good N(%)	Excellent N(%)	Mean Mean (SD)	Factors Affecting Reasons
current pelvic pain	8 (7.0)	24 (21.1)	14 (12.3)	39 (34.5)	27 (23.7)	2.5 (1.3)	-
current dyspareunia	7 (6.1)	21 (18.4)	13 (11.4)	37 (32.5)	35 (30.7)	2.6 (1.3)	-
fear of pain	17 (14.9)	22 (19.3)	22 (19.3)	37 (32.5)	15 (13.2)	2.1 (1.3)	-
fear of dyspareunia	10 (8.8)	19 (16.7)	16 (14.0)	39 (34.2)	27 (23.7)	2.5 (1.3)	Latin America ↓ Being a specialist longer ↓
longer duration	65 (57.0)	31 (27.2)	8 (7.0)	7 (6.1)	1 (0.9)	0.6 (0.9)	Number of surgeries ↓

↓ indicates that a factor is associated with negative outcomes such as “absolutely not a reason” or “mostly not a reason”.

## Data Availability

The data presented in this study are available on request from the corresponding author.
